# Critical Evaluation and Thermodynamic Re-Optimization of the Si–P and Si–Fe–P Systems

**DOI:** 10.3390/ma16114099

**Published:** 2023-05-31

**Authors:** Zhimin You, Hao Zhang, Senlin Cui, Zhouhua Jiang, In-Ho Jung

**Affiliations:** 1 School of Metallurgy, Northeastern University, No. 3-11, Wenhua Road, Heping District, Shenyang 110819, Chinajiangzh@smm.neu.edu.cn (Z.J.); 2 School of Civil Aviation, Northwestern Polytechnical University, Xi’an 710072, China; 3 Department of Materials Science and Engineering, and Research Institute of Advanced Materials (RIAM), Seoul National University, 1 Gwanak-ro, Gwanak-gu, Seoul 08826, Republic of Korea; in-ho.jung@snu.ac.kr

**Keywords:** thermodynamic modeling, Si–P system, Si–Fe–P system, FeSi_4_P_4_, thermodynamic properties, phase diagrams

## Abstract

Thermodynamic modeling of the Si–P and Si–Fe–P systems was performed using the CALculation of PHAse Diagram (CALPHAD) method based on critical evaluation of available experimental data in the literature. The liquid and solid solutions were described using the Modified Quasichemical Model accounting for the short-range ordering and Compound Energy Formalism considering the crystallographic structure, respectively. In the present study, the phase boundaries for the liquidus and solid Si phases of the Si–P system were reoptimized. Furthermore, the Gibbs energies of the liquid solution, (Fe)_3_(P,Si)_1_, (Fe)_2_(P,Si)_1_, and (Fe)_1_(P,Si)_1_ solid solutions and FeSi_4_P_4_ compound were carefully determined to resolve the discrepancies in previously assessed vertical sections, isothermal sections of phase diagrams, and liquid surface projection of the Si–Fe–P system. These thermodynamic data are of great necessity for a sound description of the entire Si–Fe–P system. The optimized model parameters from the present study can be used to predict any unexplored phase diagrams and thermodynamic properties within the Si–Fe–P alloys.

## 1. Introduction

Si has been commonly recognized as the prime candidate for solar industry applications owing to its excellent photoconductive and electrical properties [1]. However, the photovoltaic conversion efficiency and electrical conductivity of Si solar cells highly depend on the level of impurities. In order to fabricate solar-grade Si with high purity, relatively inexpensive metallurgical-grade Si and ferrosilicon alloy are often selected as raw materials for Si refinement [2]. One of the dominant challenges of such a process is to eliminate P, which needs to be controlled to as low as $1\times 10^{-5}$ wt.% to meet the requirements for solar cells [3]. Currently, several methods, including vacuum refining [4,5], directional solidification [5,6], slag refining [7,8,9], electron-beam melting [10,11], and solvent refining [12], have been employed to remove P from Si. Implementation of these processes demands a sound thermodynamic description of the Si–P and Si–Fe–P systems. 

So far, the Fe–P [13,14,15,16,17,18,19] and Fe–Si [20,21,22,23,24,25,26,27] systems have been thermodynamically modeled in many studies and were recently reoptimized by the present authors [28,29]. A complete thermodynamic modeling of the Si–P system was performed by Jung and Zhang [30] and Liang and Schmid-Fetzer [31]. However, both modeling results exhibited some discrepancies in the phase equilibria of the Si–P system and inconsistency with the ternary Si–Fe–P system. The Si–Fe–P system was thermodynamically assessed by Yan et al. [32] and Miettinen and Vassilev-Urumov [33]. The former study [32] assessed only liquid solutions using the molecular interaction volume model. In the latter assessment [33], phase equilibria and thermodynamic properties of the whole Si–Fe–P system were not well determined using the substitutional model. Therefore, a complete re-optimization of the Si–Fe–P system is necessary to obtain an accurate and self-consistent thermodynamic description of this system.

The aim of this work is to conduct a critical thermodynamic re-optimization of the Si–P and Si–Fe–P systems to develop a more accurate and reliable thermodynamic database, which is of great importance for materials engineering embracing such alloy systems. The recently optimized Fe–P [28] and Fe–Si [29] systems by the present authors were adopted in this study. The phase equilibria and thermodynamic properties of the Si–P and Si–Fe–P systems were determined based on reliable experimental data. In particular, the Gibbs energies of the liquid, solid Si, SiP(s), SiP_2_(s), FeSi_4_P_4_(s), (Fe)_3_(P,Si)_1_, (Fe)_2_(P,Si)_1_, and (Fe)_1_(P,Si)_1_ phases were carefully optimized to resolve the discrepancies left in previous thermodynamic assessments [30,31,33]. The developed thermodynamic database of the Si–Fe–P system was used to predict experimentally unexplored phase diagrams and thermodynamic properties and can be applied to process optimization of Si refining and alloy design. All the calculations were performed using FactSage 8.2 software [34]. 

## 2. Thermodynamic Models

### 2.1. Gas Phase

The gas phase of the Si–Fe–P system is a mixture of Si(g), Si_2_(g), Si_3_(g), Fe(g), P(g), P_2_(g), and P_4_(g) species. The Gibbs energy per mole of gas ($G_{T}^{gas}$) was calculated using Equation (1):

(1) $$G_{T}^{\mathrm{gas}}=\sum x_{i}\left(G_{i}^{{}^{\circ}}+RT\mathrm{In}x_{i}\right)+RT\mathrm{In}\left(f/P^{\theta }\right)$$

where $x_{i}$ is the mole fraction of species i, $G_{i}^{{}^{\circ}}$ is the molar Gibbs energy (J/mol) of species i that can be taken from the FactPS database stored in FactSage 8.2 software [34], *R* is the gas constant (=8.314 J/(mol·K)), *T* is the temperature in Kelvin (K), $P^{\theta }$ is the atmospheric pressure (=1 atm), and *f* is the gas fugacity and is identical to the gas pressure (in atm) at normal pressure.

### 2.2. Pure Elements and Stoichiometric Compounds 

The Gibbs energies of all pure liquid and solid Si, Fe, and P elements were taken from the Scientific Group Thermodata Europe (SGTE) [35] data compilation. The Gibbs energies ($G_{T}^{{}^{\circ}}$) of all intermediate stoichiometric compounds, including Fe_3_P, Fe_2_P, FeP, and FeP_2_ of the Fe–P system, Fe_2_Si, Fe_5_Si_3_, FeSi, FeSi_2_, and Fe_3_Si_7_ of the Fe–Si system, SiP, SiP_2_ of the Si–P system, and FeSi_4_P_4_ of the Si–Fe–P system were determined from their corresponding heat capacity $C_{P}$ (J/(mol·K)), standard enthalpy of formation ${∆H}_{298.15K}^{{}^{\circ}}$ (J/mol) and standard entropy $S_{298.15K}^{{}^{\circ}}$ (J/(mol·K)) as expressed by Equation (2):

(2) $$G_{T}^{{}^{\circ}}=\left(\Delta H_{298.15K}^{{}^{\circ}}+\int_{298.15K}^{T}C_{P}dT\right)-T\left(S_{298.15K}^{{}^{\circ}}+\int_{298.15K}^{T}\frac{C_{P}}{T}dT\right)$$

In the cases of pure elements and stoichiometric compounds exhibiting magnetic behavior, an additional Gibbs energy of magnetic contribution $G^{mg}$ (J/mol) will be applied. In this study, $G^{mg}$ was applied to Fe (BCC_A2, FCC_A1), Fe_3_P, and Fe_5_Si_3_ and determined from an empirical expression proposed by Inden [36] and modified by Hillert and Jarl [37]:

(3) $$G^{\mathrm{mg}}=RT\mathrm{In}\left(\beta +1\right)g(\tau )$$

where *τ* is expressed by $T/T^{*}$, $T^{*}$ is the Curie temperature $T_{C}$ (K) for ferromagnetic ordering or the Néel temperature $T_{N}$ (K) for anti-ferromagnetic ordering, g(τ) is a polynomial function that can be found elsewhere [37], and *β* is the mean magnetic moment per mole of atoms expressed in Bohr magnetons (μ_B_/mol).

### 2.3. Solid Solutions

It has been well known that Fe and P are soluble in solid Si to form a solution in diamond_A4 cubic structure. Further, Si and P can dissolve into *γ*-Fe (FCC_A1) to generate a substitutional solution. In the binary Si-Fe system, the BCC phase exhibits a long-range ordering from disordered structure (BCC_A2) to ordered structure (BCC_B2) transition [29], so this order/disorder transition was thus taken into account by the present modeling for the ternary Si–Fe–P system. Furthermore, Si was considered to substitute the P atoms of Fe_3_P, Fe_2_P, and FeP to form (Fe)_3_(P, Si)_1_, (Fe)_2_(P, Si)_1_, and (Fe)_1_(P, Si)_1_ solid solutions represented by Me_3_P, Me_2_P, and MeP, correspondingly. The Gibbs energies of all involved solid solutions in the sub-systems of Si–Fe–P were described using the Compound Energy Formalism (CEF) with consideration of their crystal structures [38]. 

#### 2.3.1. FCC_A1 and Solid Si Solutions

FCC_A1 and solid Si (diamond_A4) solutions were described with the formula (Fe, Si, P)_1_(Va)_1_, and their Gibbs energies per formula unit were calculated as follows:

(4) $$\begin{array}{c} G_{S}^{\mathrm{disorder}}=\sum_{i=\mathrm{Fe},\mathrm{Si},P}x_{i}G_{i}^{o}+RΤ\sum_{i=\mathrm{Fe},\mathrm{Si},P}x_{i}\mathrm{In}x_{i}\\ +\sum_{m=0,1,2...}x_{\mathrm{Fe}}x_{P}L_{\mathrm{Fe},P}^{m}+\sum_{k=0,1,2...}x_{\mathrm{Si}}x_{P}L_{\mathrm{Si},P}^{k}+\sum_{p=0,1,2...}x_{\mathrm{Fe}}x_{\mathrm{Si}}L_{\mathrm{Fe},\mathrm{Si}}^{p}+\sum_{q=0,1,2...}x_{\mathrm{Fe}}x_{\mathrm{Si}}x_{P}L_{\mathrm{Fe},\mathrm{Si},P}^{q}+G^{\mathrm{mg}} \end{array}$$

where $x_{i}$ is the mole fraction of component i and $G_{i}^{{}^{\circ}}$ is the molar Gibbs energy (J/mol) of pure solid i (i = Fe, Si, P), $L_{Fe,P}^{m}$, $L_{Si,P}^{k}$, $L_{Fe,Si}^{p}$, and $L_{Fe,Si,P}^{q}$ are adjustable interaction parameters of corresponding binary and ternary systems (J/mol), and $G^{mg}$ is the magnetic contribution to the Gibbs energy (J/mol). 

#### 2.3.2. Disordered/Ordered BCC Solid Solution

The Gibbs energy of the BCC solid solution was modeled by combining the disordered part described with the formula (Fe, Si, P)_1_(Va)_3_ and the ordered part with the formula (Fe, Si, P)_0.5_(Fe, Si, P)_0.5_(Va)_3_. The Gibbs energy of the disordered part can be calculated from Equation (4), while that of the ordered part can be calculated using the following equations:

(5) $$\Delta G_{\mathrm{BCC}}^{\mathrm{order}}=G_{\mathrm{BCC}}^{\mathrm{order}}(y_{i}^{\prime },y_{j}^{\prime \prime })-G_{\mathrm{BCC}}^{\mathrm{order}}{\left(y_{i}^{\prime },y_{j}^{\prime \prime }\right)}_{y_{k}^{\prime }=y_{k}^{\prime \prime }}$$

(6) $$\begin{array}{cc} G_{\mathrm{BCC}}^{\mathrm{order}}(y_{i}^{\prime },y_{j}^{\prime \prime })= & y_{\mathrm{Fe}}^{\prime }y_{\mathrm{Fe}}^{\prime \prime }G_{\mathrm{Fe}:\mathrm{Fe}}+y_{\mathrm{Si}}^{\prime }y_{\mathrm{Si}}^{\prime \prime }G_{\mathrm{Si}:\mathrm{Si}}+y_{P}^{\prime }y_{P}^{\prime \prime }G_{P:P}\\ & +y_{\mathrm{Fe}}^{\prime }y_{\mathrm{Si}}^{\prime \prime }G_{\mathrm{Fe}:\mathrm{Si}}+y_{\mathrm{Si}}^{\prime }y_{\mathrm{Fe}}^{\prime \prime }G_{\mathrm{Si}:\mathrm{Fe}}+y_{\mathrm{Fe}}^{\prime }y_{P}^{\prime \prime }G_{\mathrm{Fe}:P}\\ & +y_{P}^{\prime }y_{\mathrm{Fe}}^{\prime \prime }G_{P:\mathrm{Fe}}+y_{\mathrm{Si}}^{\prime }y_{P}^{\prime \prime }G_{\mathrm{Si}:P}+y_{P}^{\prime }y_{\mathrm{Si}}^{\prime \prime }G_{P:\mathrm{Si}}\\ & +0.5RT\left(y_{\mathrm{Fe}}^{\prime }\mathrm{In}y_{\mathrm{Fe}}^{\prime }+y_{\mathrm{Si}}^{\prime }\mathrm{In}y_{\mathrm{Si}}^{\prime }+y_{P}^{\prime }\mathrm{In}y_{P}^{\prime }\right)\\ & +0.5RT\left(y_{\mathrm{Fe}}^{\prime \prime }\mathrm{In}y_{\mathrm{Fe}}^{\prime \prime }+y_{\mathrm{Si}}^{\prime \prime }\ln y_{\mathrm{Si}}^{\prime \prime }+y_{P}^{\prime \prime }\ln y_{P}^{\prime \prime }\right)\\ & +\sum_{i,j,k}y_{i}^{\prime }y_{j}^{\prime }y_{k}^{\prime \prime }L_{i,j:k}+\sum_{i,j,k}y_{k}^{\prime }y_{i}^{\prime \prime }y_{j}^{\prime \prime }L_{k:i,j}+G^{\mathrm{mg}} \end{array}$$

where i, j, and k represent Fe, Si, P. $y_{i}^{\prime }$, $y_{j}^{\prime }$, $y_{k}^{\prime }$ and $y_{i}^{\prime \prime }$, $y_{j}^{\prime \prime }$, $y_{k}^{\prime \prime }$ are site fractions of component i, j, k in the first and second lattice of the formula (Fe, Si, P)_0.5_(Fe, Si, P)_0.5_(Va)_3_, respectively. The Gibbs energy of the BCC solid solution combining both ordered and disordered contributions was determined from Equation (7):

(7) $$G_{\mathrm{BCC}}^{\mathrm{sol}.}=G_{S}^{\mathrm{disorder}}+\Delta G_{\mathrm{BCC}}^{\mathrm{order}}$$

when the site fractions of component i in the first sublattice are equal to that in the second sublattice ($y_{i}^{\prime }=y_{i}^{\prime \prime }$), then the ordering contribution ${\Delta G}_{BCC}^{order}$ equals nil, and the Gibbs energy of the BCC phase is the same as that of the disordered BCC_A2 ($G_{S}^{disorder}$) calculated by Equation (4). In the case of $y_{i}^{\prime }\neq y_{i}^{\prime \prime }$, then ordering contribution ${\Delta G}_{BCC}^{order}$ becomes negative, and the Gibbs energy of the BCC_B2 phase can be calculated using Equation (7). 

#### 2.3.3. Other Solid Solutions (Me_3_P, Me_2_P, MeP)

The solid solutions Me_3_P, Me_2_P, and MeP were described with the formula (Fe)_n_(P, Si)_1_, where n=3,2,1 for Me_3_P, Me_2_P, and MeP, respectively. Their Gibbs energies per formula unit were calculated based on the CEF [38] as follows:

(8) $$G_{{\mathrm{Me}}_{n}P}^{\mathrm{sol}.}=y_{P}G_{{\mathrm{Fe}}_{n}P}^{{}^{\circ}}+y_{\mathrm{Si}}G_{{\mathrm{Fe}}_{n}\mathrm{Si}}+RΤ\left(y_{P}\ln y_{P}+y_{\mathrm{Si}}\ln y_{\mathrm{Si}}\right)+\sum_{m=0,1,2...}y_{P}y_{\mathrm{Si}}L_{\mathrm{Fe}:P,\mathrm{Si}}^{m}+G^{\mathrm{mg}}$$

here, $G_{{Fe}_{3}P}^{{}^{\circ}}$, $G_{{Fe}_{2}P}^{{}^{\circ}}$, and $G_{FeP}^{{}^{\circ}}$ are optimized Gibbs energies (J/mol) of stoichiometric ${Fe}_{3}P$, ${Fe}_{2}P,$ and FeP compounds of the Fe–P system [28]; $G_{{Fe}_{3}Si}$, $G_{{Fe}_{2}Si}$, and $G_{FeSi}$ are Gibbs energies (J/mol) of Fe_3_Si, Fe_2_Si, and FeSi combinations respectively, which need to be optimized in this work; $y_{P}$ and $y_{Si}$ are site fractions of P and Si in the second sublattice, respectively; $L_{Fe:P,Si}^{m}$ is the adjustable interaction parameter (J/mol), and $G^{mg}$ is the magnetic contribution to the Gibbs energy (J/mol).

### 2.4. Liquid Solution

The Gibbs energies of all liquid solutions within the Si–Fe–P system were described by the Modified Quasichemical Model (MQM) [39,40] in pair approximation. The MQM, with consideration of the bond structure in a liquid solution, gives a more realistic thermodynamic description of the liquid solution than the Bragg–Williams Random Mixing Model. In MQM, the pair formation Gibbs energy can be expressed as a polynomial in the pair fraction instead of the component fraction, and the coordination number of each component can be varied with composition to reproduce the short-range ordering more easily.

In the case of the binary A–B liquid phase, A atoms and B atoms are distributed over the quasi-lattice sites, and the following atom pair exchanging reaction is considered:

(9) $$(A-A)+(B-B)=2(A-B);\Delta g_{\mathrm{AB}}$$

where (A–A), (B–B), and (A–B) represent the first-nearest-neighbor pairs between components A and A, B and B, A and B, and $\Delta g_{AB}$ is the Gibbs energy change for the formation of 2 moles (A–B) pairs from 1 mole (A–A) pairs and 1 mole (B–B) pairs. The Gibbs energy of the A–B solution was calculated using Equation (10):

(10) $$G_{\mathrm{AB}}^{L}=(n_{A}G_{A}^{{}^{\circ}}+n_{B}G_{B}^{{}^{\circ}})-T\Delta S_{\mathrm{AB}}^{\mathrm{conf}.}+n_{\mathrm{AB}}(\Delta g_{\mathrm{AB}}/2)$$

where $n_{A}$ and $n_{B}$ are the mole numbers of A atoms and B atoms (mol), $G_{A}^{{}^{\circ}}$ and $G_{B}^{{}^{\circ}}$ are the molar Gibbs energies of pure A and B in a liquid state (J/mol), and ${\Delta S}_{AB}^{conf.}$ is the configurational entropy of mixing (J/(mol·K)) given by Equation (11):

(11) $$\Delta S_{\mathrm{AB}}^{\mathrm{conf}.}=-R(n_{A}\ln X_{A}+n_{B}\ln X_{B})-R\left[n_{\mathrm{AA}}\ln\left(\frac{X_{\mathrm{AA}}}{Y_{A}^{2}}\right)+n_{\mathrm{BB}}\ln\left(\frac{X_{\mathrm{BB}}}{Y_{B}^{2}}\right)+n_{\mathrm{AB}}\ln\left(\frac{X_{\mathrm{AB}}}{2Y_{A}Y_{B}}\right)\right]$$

where $n_{AA}$, $n_{BB}$, and $n_{AB}$ represent the mole numbers of (A–A), (B–B) and (A–B) pairs (mol), $X_{AA}$, $X_{AB}$, and $X_{AB}$ are pair fractions of corresponding atom pairs, and $Y_{A}$ and $Y_{B}$ are coordination equivalent fractions of A atoms and B atoms. The pair fractions $X_{AA}$, $X_{BB}$, $X_{AB}$ and coordination equivalent fractions $Y_{A}$, $Y_{B}$ can be calculated using Equations (12)–(16):

(12) $$X_{\mathrm{AA}}=n_{\mathrm{AA}}/\left(n_{\mathrm{AA}}+n_{\mathrm{AB}}+n_{\mathrm{BB}}\right)$$

(13) $$X_{\mathrm{AB}}=n_{\mathrm{AB}}/\left(n_{\mathrm{AA}}+n_{\mathrm{AB}}+n_{\mathrm{BB}}\right)$$

(14) $$X_{\mathrm{BB}}=n_{\mathrm{BB}}/\left(n_{\mathrm{AA}}+n_{\mathrm{AB}}+n_{\mathrm{BB}}\right)$$

(15) $$Y_{A}=X_{\mathrm{AA}}+\frac{1}{2}X_{\mathrm{AB}}$$

(16) $$Y_{B}=X_{\mathrm{BB}}+\frac{1}{2}X_{\mathrm{AB}}$$

${\Delta g}_{AB}$ in Equations (9) and (10) is the model parameter for reproducing the Gibbs energy of the A–B liquid solution (J/mol) and can be expanded as a polynomial in terms of the atomic pair fractions $X_{AA}$ and $X_{BB}$.

(17) $$\Delta g_{\mathrm{AB}}=\Delta g_{\mathrm{AB}}^{{}^{\circ}}+\sum_{i\geq 1}g_{\mathrm{AB}}^{i0}X_{\mathrm{AA}}^{i}+\sum_{j\geq 1}g_{\mathrm{AB}}^{0j}X_{\mathrm{BB}}^{j}$$

where ${\Delta g}_{AB}^{{}^{\circ}}$, $g_{AB}^{i0}$ and $g_{AB}^{0j}$ are the adjustable model parameters (J/mol) that can be functions of the temperature. In the MQM, the coordination numbers of A and B, and $Z_{A}$ and $Z_{B}$, can be varied with the composition to reproduce the short-range ordering.

(18) $$\frac{1}{Z_{A}}=\frac{1}{Z_{\mathrm{AA}}^{A}}\left(\frac{2n_{\mathrm{AA}}}{2n_{\mathrm{AA}}+n_{\mathrm{AB}}}\right)+\frac{1}{Z_{\mathrm{AB}}^{A}}\left(\frac{n_{\mathrm{AB}}}{2n_{\mathrm{AA}}+n_{\mathrm{AB}}}\right)$$

(19) $$\frac{1}{Z_{B}}=\frac{1}{Z_{\mathrm{BB}}^{B}}\left(\frac{2n_{\mathrm{BB}}}{2n_{\mathrm{BB}}+n_{\mathrm{AB}}}\right)+\frac{1}{Z_{\mathrm{BA}}^{B}}\left(\frac{n_{\mathrm{AB}}}{2n_{\mathrm{BB}}+n_{\mathrm{AB}}}\right)$$

here $Z_{AA}^{A}$ is the value $Z_{A}$ when all nearest neighbors of an A atom are A atoms, and $Z_{AB}^{A}$ is the value of $Z_{A}$ when all nearest neighbors of the A atom are B atoms. $Z_{BB}^{B}$ and $Z_{BA}^{B}$ are defined in an analogous manner. In the present study, $Z_{FeFe}^{Fe}=Z_{SiSi}^{Si}=Z_{PP}^{P}=6$ [28,29], $Z_{PFe}^{P}=Z_{PSi}^{P}=Z_{FeSi}^{Fe}=Z_{SiFe}^{Si}=Z_{SiP}^{Si}=6$ [28,29,30], and $Z_{FeP}^{Fe}=3$ [28], as listed in Table 1. 

The Gibbs energy of the ternary Si–Fe–P liquid solution can be predicted from the interpolation of Gibbs energies of its sub-binary systems based on their nature. In the present study, an asymmetric “Toop-like” geometric interpolation [40] with Fe as the “asymmetric component” was used for the Si–Fe–P system since the Fe–P and Fe–Si liquid solutions show much more negative deviations from the Si–P liquid solution. Based on this interpolation, the entropy of mixing and Gibbs energy of the ternary Si–Fe–P liquid solution were calculated using Equations (20) and (21):

(20) $$\Delta S_{\mathrm{Si},\mathrm{Fe},P}^{\mathrm{conf}.}=-R\sum_{i=\mathrm{Si},\mathrm{Fe},P}n_{i}\ln X_{i}-R\left[\sum_{j=\mathrm{Si},\mathrm{Fe},P}n_{\mathrm{jj}}\left(\frac{X_{\mathrm{jj}}}{Y_{j}^{2}}\right)+\sum_{k,m=\mathrm{Si},\mathrm{Fe},P}^{k\neq m}n_{\mathrm{km}}\ln\left(\frac{X_{\mathrm{km}}}{2Y_{k}Y_{m}}\right)\right]$$

(21) $$G_{\mathrm{Si},\mathrm{Fe},P}^{L}=\sum_{i=\mathrm{Si},\mathrm{Fe},P}n_{i}G_{i}^{{}^{\circ}}-T\Delta S_{\mathrm{Si},\mathrm{Fe},P}^{\mathrm{conf}.}+\sum_{j,k=\mathrm{Si},\mathrm{Fe},P}^{j\neq k}\left(n_{\mathrm{jk}}/2\right)\Delta g_{\mathrm{jk}}$$

here, ${\Delta g}_{jk}$ (j,k=Si,Fe,P) is the pair formation Gibbs energy depending on the thermodynamic symmetry of the ternary system. ${\Delta g}_{FeP}$ and ${\Delta g}_{FeSi}$ for the asymmetric Fe–P and Fe–Si systems are respectively calculated using Equations (22) and (23):

(22) $$\Delta g_{\mathrm{FeSi}}=\Delta g_{\mathrm{FeSi}}^{{}^{\circ}}+\sum_{(i+j)\geq 1}g_{\mathrm{FeSi}}^{\mathrm{ij}}x_{\mathrm{FeFe}}^{i}{\left(x_{\mathrm{SiSi}}+x_{\mathrm{SiP}}+x_{\mathrm{PP}}\right)}^{j}+\sum_{i\geq 0,j\geq 0,k\geq 1}g_{\mathrm{FeSi}(P)}^{\mathrm{ijk}}x_{\mathrm{FeFe}}^{i}{\left(x_{\mathrm{SiSi}}+x_{\mathrm{SiP}}+x_{\mathrm{PP}}\right)}^{j}{\left(\frac{Y_{P}}{Y_{\mathrm{Si}}+Y_{P}}\right)}^{k}$$

(23) $$\Delta g_{\mathrm{FeP}}=\Delta g_{\mathrm{FeP}}^{{}^{\circ}}+\sum_{(i+j)\geq 1}g_{\mathrm{FeP}}^{\mathrm{ij}}x_{\mathrm{FeFe}}^{i}{\left(x_{\mathrm{SiSi}}+x_{\mathrm{SiP}}+x_{\mathrm{PP}}\right)}^{j}+\sum_{i\geq 0,j\geq 0,k\geq 1}g_{\mathrm{FeP}(\mathrm{Si})}^{\mathrm{ijk}}x_{\mathrm{FeFe}}^{i}{\left(x_{\mathrm{SiSi}}+x_{\mathrm{SiP}}+x_{\mathrm{PP}}\right)}^{j}{\left(\frac{Y_{\mathrm{Si}}}{Y_{\mathrm{Si}}+Y_{P}}\right)}^{k}$$

while ${\Delta g}_{SiP}$ for the symmetric Si–P system was calculated using Equation (24):

(24) $$\Delta g_{\mathrm{SiP}}=\Delta g_{\mathrm{SiP}}^{{}^{\circ}}+\sum_{(i+j)\geq 1}g_{\mathrm{SiP}}^{\mathrm{ij}}{\left(\frac{x_{\mathrm{SiSi}}}{x_{\mathrm{SiSi}}+x_{\mathrm{SiP}}+x_{\mathrm{PP}}}\right)}^{i}{\left(\frac{x_{\mathrm{PP}}}{x_{\mathrm{SiSi}}+x_{\mathrm{SiP}}+x_{\mathrm{PP}}}\right)}^{j}+\sum_{i\geq 0,j\geq 0,k\geq 1}g_{\mathrm{SiP}(\mathrm{Fe})}^{\mathrm{ijk}}{\left(\frac{x_{\mathrm{SiSi}}}{x_{\mathrm{SiSi}}+x_{\mathrm{SiP}}+x_{\mathrm{PP}}}\right)}^{i}{\left(\frac{x_{\mathrm{PP}}}{x_{\mathrm{SiSi}}+x_{\mathrm{SiP}}+x_{\mathrm{PP}}}\right)}^{j}Y_{\mathrm{Fe}}^{k}$$

where $g_{FeSi}^{ij}$, $g_{FeP}^{ij}$, and $g_{SiP}^{ij}$ are binary liquid model parameters (J/mol) and $g_{FeSi(P)}^{ijk}$, $g_{FeP(Si)}^{ijk}$, and $g_{SiP(Fe)}^{ijk}$ are ternary liquid model parameters (J/mol). 

## 3. Critical Evaluation and Thermodynamic Optimization

Thermodynamic optimization of the Si–P and Si–Fe–P systems was performed using the CALPHAD approach based on the critical evaluation of all available phase equilibria and thermodynamic property data. The liquid and solid solutions of all sub-systems were modeled using the MQM [39,40] and CEF [38], respectively. The optimized model parameters of the Si-Fe-P system are summarized in Table 1 and the crystal structure information of all solid phases of this system in Table 2.

### 3.1. The Si–P System

The Si–P system has been well-reviewed by Mostafa [42], Jung and Zhang [30], and Liang and Schmid-Fetzer [31]. According to the literature, liquid solution, solid Si (diamond_A4), red P, SiP, and SiP_2_ are stable condensed phases in the Si–P system and are also adopted by the present study. 

#### 3.1.1. Phase Diagram

**Figure 1:**
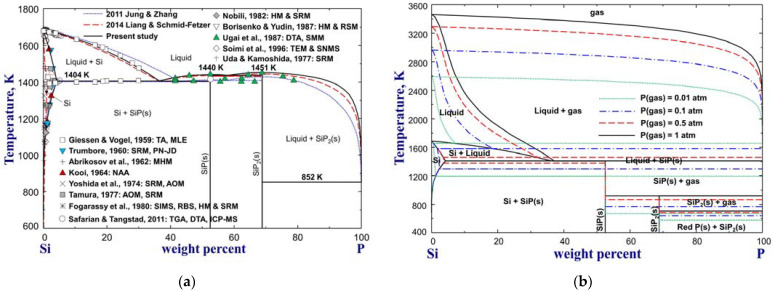
The phase diagrams of the Si–P system (**a**) with suppression of gas phase and (**b**) with gas phase at a total pressure of 0.01, 0.1, 0.5, and 1 atm, compared to available experimental data [43,44,45,46,47,48,49,50,51,52,53,54,55]. For abbreviations, see Abbreviations Section.

The calculated Si–P phase diagrams from previous assessments [30,31] and the present study are plotted in Figure 1, along with the experimental data [43,44,45,46,47,48,49,50,51,52,53,54,55]. The phase diagrams with suppression of the gas phase are plotted in Figure 1a, and those with gas phase in a total pressure of 0.01, 0.1, 0.5, and 1 atm are in Figure 1b. In the thermodynamic assessment by Jung and Zhang [30], a constant MQM parameter was used to describe the Si–P liquid solution; however, their calculation of liquidus boundaries shows some deviations from the experimental results of the particularly high-P region [43,55]. The assessed eutectic composition (wt.%P=42.9) for the reaction liquid=Si+SiP(s) in their study was found to be much overestimated, which is why the liquidus data nearing the SiP compound could not be reproduced. As a result, SiP was calculated to melt congruently at 1410 K to reproduce the data (1412 ± 2 K) suggested by Safarian and Tangstad [54]. However, the reliability of this data is doubtful for given reasons [31]. In the subsequent assessment by Liang and Schmid-Fetzer [31] using the substitutional solution model, the eutectic reaction liquid=Si+SiP(s) was modified to occur at wt.%P=36.6 and 1404 K, and the melting points of SiP and SiP_2_ were increased respectively to 1434 K and 1443 K to bridge the gap with the experimental data of Ugai et al. [55]. These data were measured from samples in the actual composition range of the compounds and considered more reliable. Compared to the assessment of Jung and Zhang [30], the liquidus boundaries calculated by Liang and Schmid-Fetzer [31] show certain improvements, as shown in Figure 1a. The eutectic composition and temperature proposed by Liang and Schmid-Fetzer [31] were inherited by the present study. Meanwhile, the liquidus temperatures were increased to fit better with the experimental results [43,55]. In particular, the melting temperatures of SiP and SiP_2_ were further optimized to 1440 K and 1451 K, respectively, to match the data of Ugai et al. [55]. However, it is worth noting that the discrepancies between the solidus and liquidus temperatures in the region of SiP to SiP_2_ could not be resolved [30,31]. The eutectic reaction $Liquid=SiP\left(s\right)+{SiP}_{2}(s)$ was reported by Ugai et al. [55] to occur at 1398 K, which is 53 K lower than the melting point of SiP_2_. If such a big difference was constrainedly reconciled, then P-rich side parameters with high-temperature dependence had to be introduced to the liquid phase. This would, unfortunately, result in a “two-liquid phase” immiscibility gap at very high temperatures and also conflicts with phase equilibria of the Si-rich region. In the present study, the eutectic temperature was determined to be 1436 K, exhibiting a difference of 15 K from the SiP_2_ melting point.

**Figure 2:**
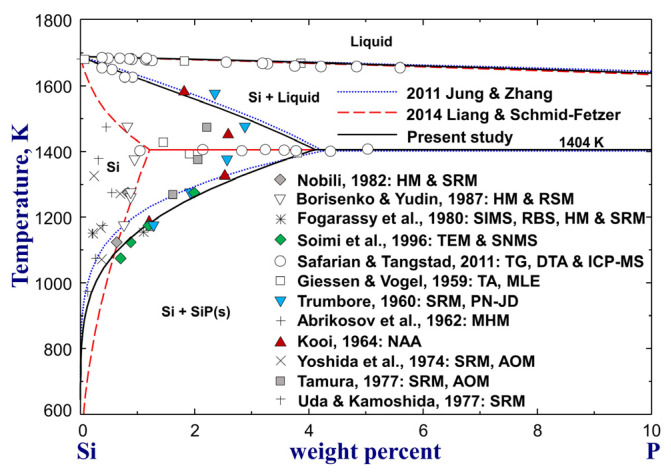
The calculated Si–P phase diagrams of the Si-rich region, along with experimental data [43,44,45,46,47,48,49,50,51,52,53,54]. For abbreviations, see Abbreviations Section.

Figure 2 shows calculated Si–P phase diagrams of the Si-rich region compared to experimental data [43,44,45,46,47,48,49,50,51,52,53,54]. Since these phase diagram data have been reviewed previously [30,31,42], it is not necessary to review them again in the present study. The solubility data for P in solid Si are scattered significantly below 1473 K, as shown in Figure 2. It is hard to judge the accuracy of these data just from their experimental techniques. In the modeling of Liang and Schmid-Fetzer [31], more weight was given to the solidus data of Safarian and Tangstad [54] at eutectic temperature and solvus data of Nobili [51] and Borisenko and Yudin [52] in the determination of solid Si boundary. As a result, a maximum solubility of wt.%P = 1.2 in Si at the eutectic temperature (1404 K) was calculated. According to Jung and Zhang [30], a much higher P solubility limit of wt.%P = 4.2 at 1400 K was calculated to reproduce the higher-temperature solidus data of Trumbore [44], Kooi [46], and Safarian and Tangstad [54], which show good consistency within experimental errors. From a thermodynamic modeling point of view, it is not possible to reproduce the high-temperature and low-temperature solidus data of Safarian and Tangstad [54] simultaneously. The inconsistency between these data [54] was not clarified in both previous assessments. After careful examination of their experiments [54], it was found that mass loss happened continuously, even after the dissociation of SiP, due to the evaporation of P from the samples. Therefore, delayed chemical analysis after TG/DSC analysis would lead to an underestimation of the P content of the solid Si phase. In the present optimization, the Si phase boundaries above the eutectic temperature by Jung and Zhang [30] were adopted with slight modification, while the boundaries below the eutectic temperature were pushed to the P-richer side using a much negative interaction parameter $L_{Si,P:Va}^{Diamond\_A4}=-50208$ J/mol (Table 1) to reproduce the experimental data of Trumbore [44], Kooi [46], and Soimi et al. [53], which are in good consistency, as shown in Figure 2. 

The distribution coefficient of P in Si, represented by $L_{P}=C_{P,S}/C_{P,L}$ ($C_{P,S}$ and $C_{P,L}$: concentration of P in solid and liquid Si, respectively), is a key factor in describing the behavior of P during the crystallizing and melting of Si. The equilibrium $L_{P}$ was reported to be 0.038 by Hall [56] based on conductivity measurements, 0.09 by Safarian and tangstad [54] from the solidus and liquidus boundaries of Si–P alloys, and 0.123 by Borisenko and Yudin [52] from the enthalpy change of P in solid and liquid Si phases. A much higher distribution coefficient ($L_{P}=0.35$) was originally obtained by Struthers [57] by means of the Czochralski crystal growth and radiochemical analysis. This value was cited as the equilibrium distribution coefficient in various literature [44,58,59,60,61], which was even mistaken as “new” sources of experimental data [5,54,62]. Huff et al. [62] investigated the distribution coefficient of P in Si under different Czochralski crystal pulling rates. As a result, an “effective” distribution coefficient $L_{P}=0.32$ at the pulling rate of 1.1 mils/sec and $L_{P}=0.42$ at the pulling rate of 1.8 mils/sec, were derived. According to Huff et al. [62], the effective $L_{P}$ is dependent on the crystal growth rate, impurity concentration, interface orientation, temperature gradients, stirring conditions, etc. Recently, Li et al. [6] determined the effective $L_{P}=0.31$ and 0.33 from directional solidification ingots grown at the rate of ${2.08\times 10}^{-6}$ m/s and ${3.08\times 10}^{-6}$ m/s, respectively. It is noticeable that $L_{P}$ values resulting from the Czochralski crystal growth method [57,62] and the directional solidification method [6] are typically higher than those from conductivity and thermochemical equilibrium measurements [52,54,56]. Because such Czochralski crystal growth and directional solidification experiments [6,57,62] proceeded typically at non-equilibrium conditions depending on the Czochralski rate and temperature gradient, so very slight supercooling of dilute Si–P alloys can lead to distinct concentration of P in the primary Si crystals. Therefore, these higher $L_{P}$ values, which are “so-called” effective distribution coefficients, should be overestimated compared to equilibrium ones. The equilibrium $L_{P}$ can be determined from the solidus and liquidus boundaries depending on the temperature. Based on the optimized Si–P phase diagram, as presented above, $L_{P}$ increases with the decline in temperature and was determined to be 0.06 at the Si melting temperature (1686.95 K) and 0.11 at the eutectic temperature (1404 K).

#### 3.1.2. Thermodynamic Stability of Si Phosphides

**Figure 3:**
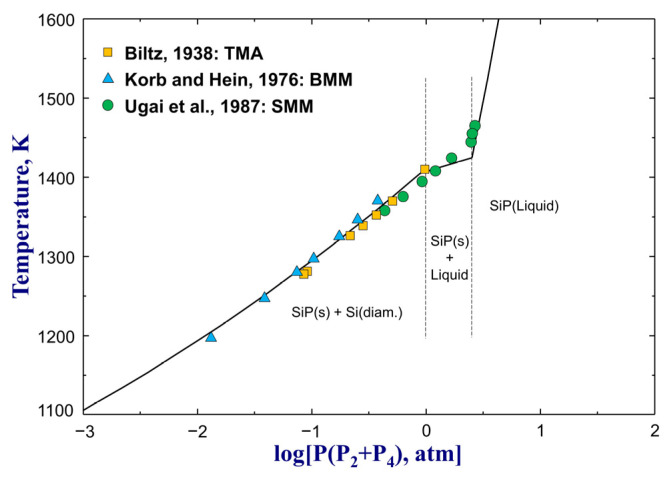
The optimized dissociation pressure (P_2_(g) + P_4_(g)) of SiP from the present study in comparison with experimental data [55,64,65]. For abbreviations, see Abbreviations Section.

The Gibbs energies of intermediate silicon phosphides SiP and SiP_2_, which were calculated from their heat capacity ($C_{P}$), standard enthalpy of formation (${∆H}_{298.15K}^{{}^{\circ}}$), and standard entropy ($S_{298.15K}^{{}^{\circ}}$) as expressed by Equation (2), were reoptimized in this study to improve the thermodynamic description of the Si–P system. The $C_{P}$ of SiP and SiP_2_ were directly taken from the assessment of Jung and Zhang [30]. Ugai et al. [63] determined $S_{298.15K}^{{}^{\circ}}=34.78$ J/(mol·K) for SiP through its low-temperature $C_{P}$ data. This value was adopted by the present study without modification. The standard enthalpy of formation for SiP was optimized to −64,000 J/mol to reproduce its melting point data from Ugai et al. [55], as shown in Figure 1a, and SiP dissociation pressure data [55,64,65] presented in Figure 3. On the other hand, since no reliable Gibbs energy data of SiP_2_ are available in the literature, so ${∆H}_{298.15K}^{{}^{\circ}}$ and $S_{298.15K}^{{}^{\circ}}$ for SiP_2_ were optimized to be −79,950 J/mol and 64 J/(mol·K), respectively, in the present study to reproduce its melting point data [55] and make SiP_2_ stable down to the ice point. The optimized thermodynamic properties of SiP and SiP_2_ are given in Table 1. 

#### 3.1.3. Thermodynamic Properties of the Si–P Liquid Solution

Thermodynamic properties of the Si–P liquid solution at any designated composition and temperature can be predicted from the developed Si–P database. As solar-grade Si demands super high purity with P content down to $10^{-5}$ wt.% level, so much attention has been paid to the thermodynamic properties of the dilute region because it is of primary importance to the Si refining process. Based on the present thermodynamic optimization, the Henrian activity coefficient of P in Si(l), $\gamma_{PinSi(l)}^{{}^{\circ}}$, depending on the temperature, was determined:

(25) $$ln\gamma_{PinSi(l)}^{{}^{\circ}}=-\frac{4025}{T}+0.7548,1687K<T<2173K$$

where *T* is the temperature in Kevin (K). Based on the optimized $\gamma_{PinSi(l)}^{{}^{\circ}}$, the molar Gibbs energy for the dissolution of P_2_(g) into liquid Si (1 wt.% standard state) was determined as follows:

(26) $$0.5P_{2}\left(g\right)=P_{inSi\left(l\right)}(wt.\%);\;{∆G}_{T}^{{}^{\circ}}=-92455+17.875T\;(J/\mathrm{mol});\;1687K<T<2173K$$

**Figure 4:**
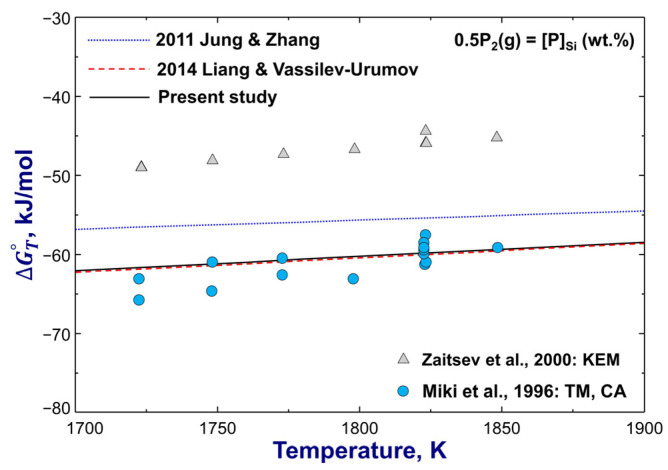
Calculated standard molar Gibbs energy change of the reaction $0.5P_{2}\left(g\right)=P(wt.\%)$, compared to experimental data [66,67]. For abbreviations, see Abbreviations Section.

The calculated Gibbs energies of P_2_(g) dissolution in Si(l) from previous assessments and the present study are plotted in Figure 4, along with the experimental data of Miki et al. [66] measured using the transportation method and those of Zaitsev et al. [67] with the Knudsen effusion mass spectrometry. These two sets of data show a distinct difference of about 15 kJ/mol. As has been pointed out [31], the activity data of Zaitsev et al. [67] are less self-consistent. Thus, their Gibbs energy data from the same experiments were not considered in the present thermodynamic modeling either. Instead, the experimental data of Miki et al. [66] were favored by the present study, which agrees with the calculation result of Liang and Vassilev-Urumov [31]. However, the modeling result of Jung and Zhang [30] shows a large deviation from both sets of experimental data [66,67], as shown in Figure 4.

**Figure 5:**
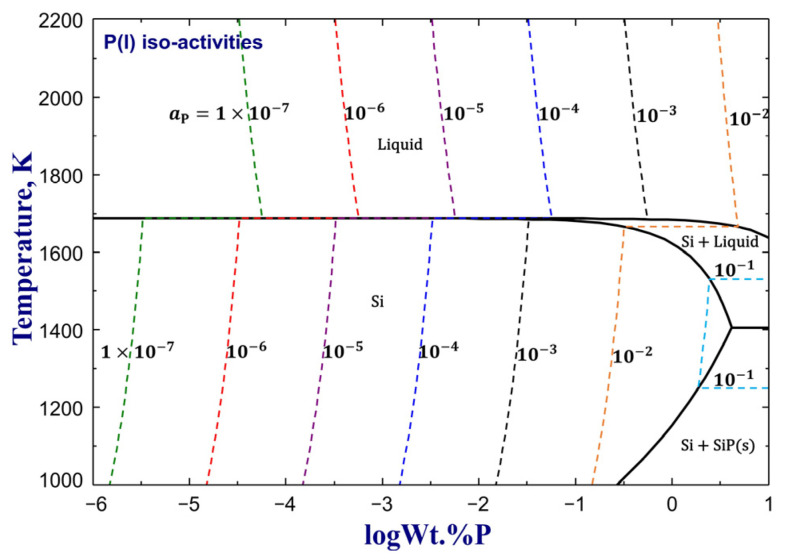
Predicted iso-activity contours of the Si–P system for $a_{P(l)}={1\times 10}^{-7}\sim {1\times 10}^{-1}$(pure P(l) as the reference state).

Based on the optimized model parameters of the Si–P system, the iso-activity contours of P in a pure liquid standard state between 1000 K and 2200 K are predicted in Figure 5. The iso-activities at $a_{P(l)}={1\times 10}^{-7}$, ${1\times 10}^{-6}$, ${1\times 10}^{-5}$, ${1\times 10}^{-4}$, ${1\times 10}^{-3}$, ${1\times 10}^{-2}$, and ${1\times 10}^{-1}$ are plotted for P(l). According to the present optimization, $a_{P(l)}$ has to be no more than ${1.99\times 10}^{-8}$ and ${1.87\times 10}^{-7}$ to control P in liquid Si within ${1\times 10}^{-5}wt.\%$ at 1773 K and ${1\times 10}^{-4}wt.\%$ at 1723 K, respectively. 

### 3.2. The Fe–P and Fe–Si Systems

The Fe–P and Fe–Si systems were recently optimized by the present authors [28,29]. In the modeling, gas phase, liquid, BCC_A2, FCC_A1, Fe_3_P, Fe_2_P, FeP, and FeP_2_ of the Fe–P system and liquid, BCC_A2, BCC_B2, FCC_A1, solid Si, Fe_2_Si, Fe_5_Si_3_, FeSi, FeSi_2_, and Fe_3_Si_7_ of the Fe–Si system were taken into account. The optimized model parameters of all these phases can be found elsewhere [28,29] and were adopted in this study. 

### 3.3. The Si–Fe–P Systems

Based on thermodynamic descriptions of the binary Fe-P, Fe–Si, and Si–P systems, gas phase, red P, liquid solution, solid solutions including FCC_A1, BCC_A2, BCC_B2, and solid Si, and stoichiometric compounds including SiP, SiP_2_, FeP_2_, Fe_2_Si, Fe_5_Si_3_, FeSi, FeSi_2_, and Fe_3_Si_7_ were treated as stable phases in the Si–Fe–P system. Moreover, the dissolution of Si in Fe_3_P and Fe_2_P to form Me_3_P and Me_2_P solid solutions in the formulas of (Fe)_3_(P, Si) and (Fe)_2_(P, Si), which have been confirmed in the experiments [68,69,70], were also considered in current thermodynamic modeling. Meanwhile, the MeP solid solution in the formula of (Fe)(P, Si), which was assumed in the assessment of Miettinen and Vassilev-Urumov [33], and the ternary FeSi_4_P_4_ compound [71,72,73] were taken into account as well. The model parameters of all binary sub-systems were combined to describe the ternary Si–Fe–P system.

#### 3.3.1. Phase Diagram

**Figure 6:**
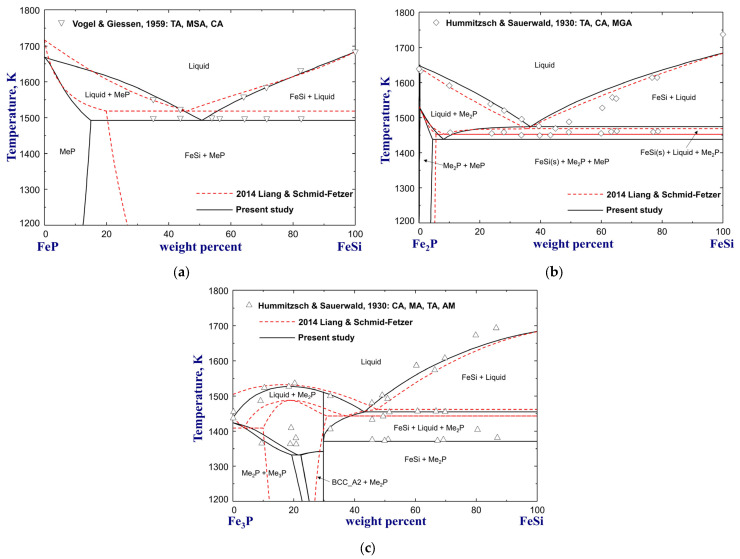
Calculated phase diagrams for the (**a**) FeP–FeSi, (**b**) Fe_2_P–FeSi, and (**c**) Fe_3_P–FeSi pseudobinary systems, compared to experimental data [71,74]. For abbreviations, see Abbreviations Section.

**Figure 7:**
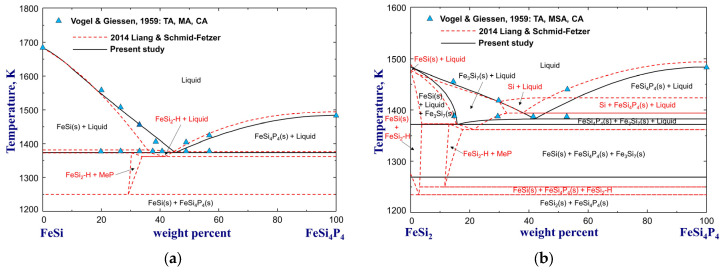
Calculated phase diagrams for the (**a**) FeSi–FeSi_4_P_4_ and (**b**) FeSi_2_–FeSi_4_P_4_ pseudobinary systems, compared to experimental data [71]. For abbreviations, see Abbreviations Section.

**Figure 8:**
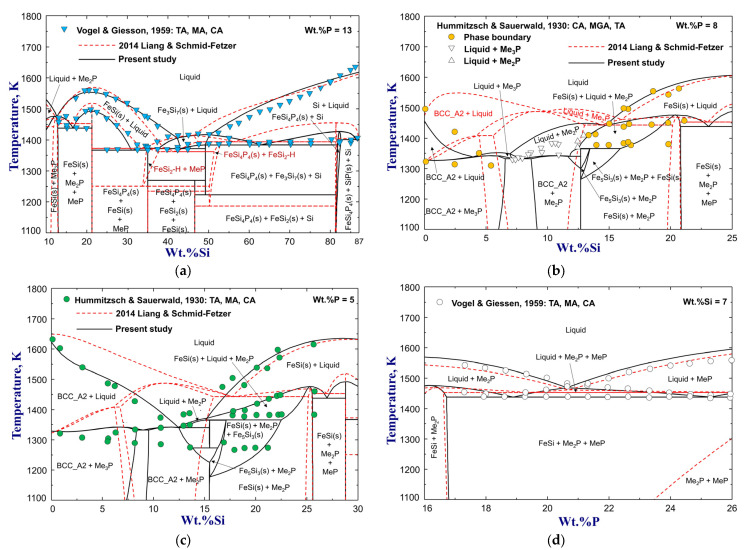
Calculated isopleths for (**a**) wt.%P=13 (**b**) wt.%P=8, (**c**) wt.%P=5, and (**d**) wt.%Si=7 of the Si–Fe–P system, compared to experimental data [71,74]. For abbreviations, see Abbreviations Section.

The phase equilibria for various vertical sections of Si–Fe–P alloys were measured by Vogel and Giessen [71] and Hummitzsch and Sauerwald [74] using thermal analysis, microscopic examination, and chemical analysis. In the former experiments [71], a full composition range of FeSi alloys containing P up to wt.%P=32.5 was used, and a ternary stoichiometric compound FeSi_4_P_4_ was found to melt at 1483 K. The experimental data for pseudobinary diagrams of FeP–FeSi, Fe_2_P–FeSi, Fe_3_P–FeSi, FeSi–Fe_4_Si_4_P_4_, FeSi_2_–FeSi_4_P_4_, and isopleths of wt.%P=13,8,5 and wt.%Si=7 are compared with the previous and present calculations in Figure 6, Figure 7 and Figure 8. Basically, the calculation results of Miettinen and Vassilev-Urumov [33] are in less satisfactory agreement with most of the phase diagram data. The discrepancies in the eutectic reaction Liquid=MeP+FeSi(s) of the FeP–FeSi section and solidus boundaries of the Fe_3_P–FeSi section have been resolved by the present study, as shown in Figure 6a,c, with careful optimization in the Gibbs energies of liquid, MeP, Me_2_P, and Me_3_P phases. Moreover, the ${∆H}_{298.15K}^{{}^{\circ}}$ and $S_{298.15K}^{{}^{\circ}}$ of FeSi_4_P_4_ compound were determined to be −33,4600 J/mol and 175 J/(mol·K) to reproduce its melting point [71] and improve the phase equilibria of the isopleth at wt.%P=13, as shown in Figure 7 and Figure 8a. In particular, the present optimization shows significant improvement in the liquidus and solidus boundaries of the low-Si region for wt.%P=5 and wt.%P=8 isopleths, compared to the previous assessment [33], as shown in Figure 8b,c. 

**Figure 9:**
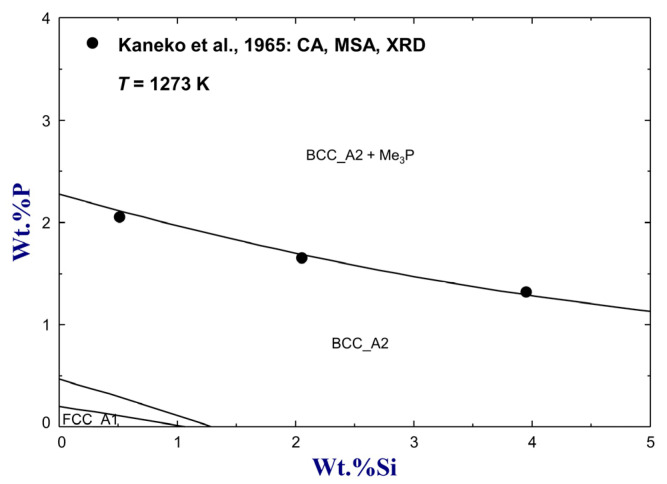
Isothermal diagram of the Si–Fe–P system on the Fe-rich corner at 1273 K, compared to experimental data [75]. For abbreviations, see Abbreviations Section.

The solubility of P in *α*-Fe (BCC_A2) with the addition of Si up to 4 wt.% at 1273 K was investigated by Kaneko et al. [75] using chemical analysis and X-ray diffraction. Their experimental data are compared to the present calculation in Figure 9. The solubility data are accurately reproduced using one interaction parameter $L_{Si,P:Va}^{BCC\_A2}=-52,300$ J/mol, as shown in Table 1. Based on the present optimization, the solubility of P in Fe–Si alloys equilibrated Me_3_P was calculated to decrease from 2.28 wt.% to 1.13 wt.% with added Si increasing up to 5 wt.%, as shown in Figure 9. 

**Figure 10:**
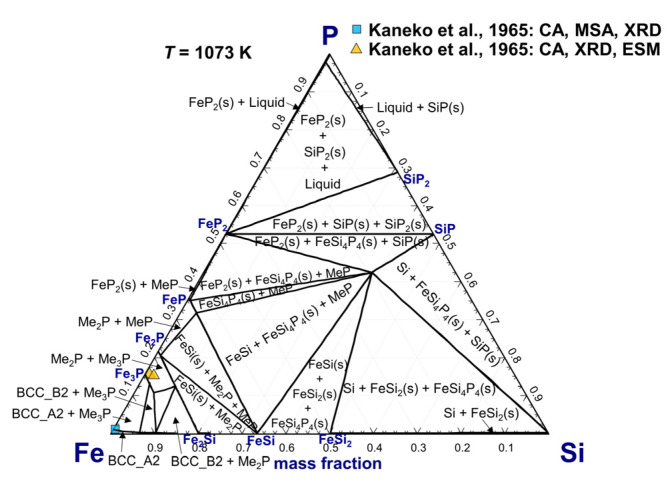
Calculated isothermal section of the Si–Fe–P phase diagram at 1073 K, compared to experimental data [75,76]. For abbreviations, see Abbreviations Section.

The Ni_3_P-type iron phosphide containing Si up to 2.1 wt.% was detected in heat-treated Si steel at 1073 K by Kaneko et al. [76] using the XRD and electrolytic separation method. Figure 10 shows the calculated isothermal section of the Si–Fe–P phase diagram at 1073 K in comparison with experimental data [75,76]. Based on the present optimization, a maximum solubility of wt.%Si=4.5 in Fe_3_P through substitution of P at 1073 K was calculated using an additional model parameter $G_{Fe:Si}^{{Me}_{3}P}=3G_{Fe(BCC)}^{{}^{\circ}}+G_{Si(Diamond\_A4)}^{{}^{\circ}}-94,500$ J/mol, as listed in Table 1. On the other hand, Me_2_P and MeP in the formulas of (Fe)_2_(P, Si) and (Fe)(P, Si) were optimized to be complete solid solutions to reproduce other phase diagram data in Figure 8, Figure 9 and Figure 10. 

**Figure 11:**
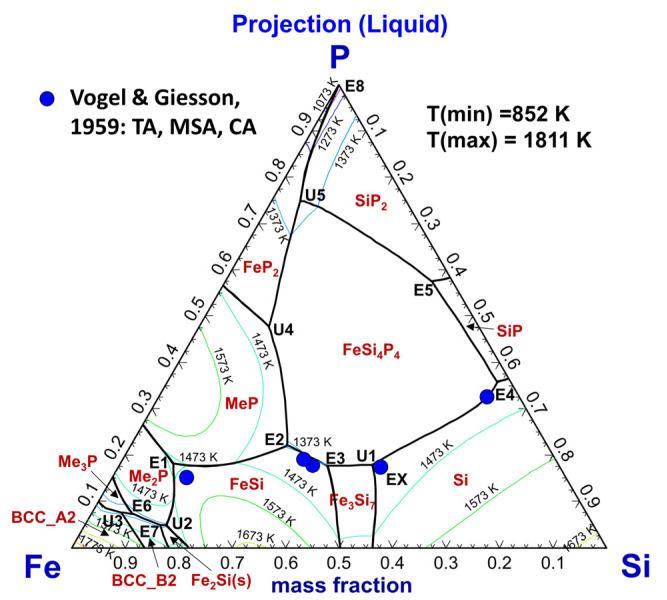
Calculated liquidus surface projection of the Fe–Si–P system between 1073 K and 1873 K, compared to experimental data [71]. For abbreviations, see Abbreviations Section.

The predicted liquidus surface projection of the Si–Fe–P system between 1073 K and 1873 K is presented in Figure 11, along with experimental data of Vogel and Giesson [71]. The invariant reactions are summarized in Table 3. The liquidus isothermals from 1773 K to 1073 K were plotted in colorful lines. The calculated invariant points **E1**, **E2**, **E3,** and **E4** agree reasonably with the data within experimental errors. However, the eutectic reaction $Liquid=Si+{Fe}_{3}{Si}_{7}(s)+{FeSi}_{4}P_{4}(s)$ in point **EX** proposed by Vogel and Giessen [71] was supposed to be a mistake and has been corrected to the peritectic reaction $Liquid+Si={Fe}_{3}{Si}_{7}(s)+{FeSi}_{4}P_{4}(s)$, which is labeled as **U1** in Figure 11.

#### 3.3.2. Thermodynamic Properties of the Si–Fe–P Liquid Solution

The optimized Gibbs energies of binary Si–P, Fe-P, and Fe–Si liquid solutions were interpolated using a “Toop-like” approximation (Fe as the asymmetric component) to describe the ternary Si–Fe–P liquid solution, as discussed in Section 2.4. Moreover, three small MQM parameters, as given in Table 1, are still necessary to reproduce the phase diagram and thermodynamic property data simultaneously. 

**Figure 12:**
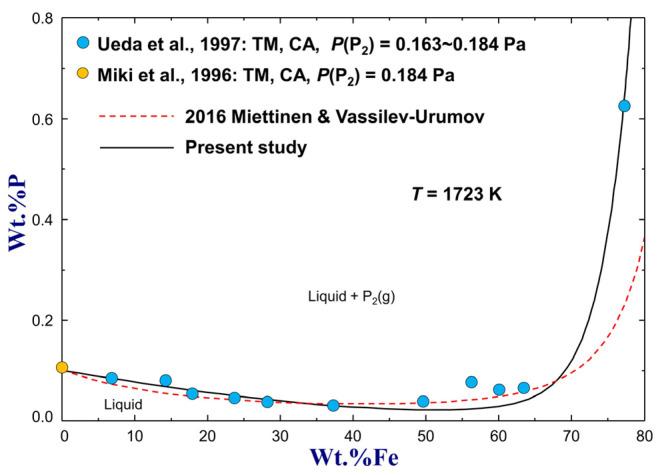
Calculated P solubility in various molten Si-Fe alloys at $P_{P_{2}(g)}=0.163\sim 0.184$ Pa and T=1723 K, compared to experimental data [66,77]. For abbreviations, see Abbreviations Section.

The solubility of P in a wide composition range of molten Si–Fe alloys was measured at 0.163~0.184 Pa of P_2_(g) pressure and 1723 K by Ueda et al. [77] using the transportation method and chemical analysis. Their experimental data, together with the P solubility data in Si(l) by Miki et al. [66], are compared to the previous assessment and present optimization results in Figure 12. As shown in the figure, the solubility of P in liquid Si decreases slightly first and then increases sharply with the increase of Fe content, and the minimum P solubility was calculated to be wt.%P=0.0212 at wt.%Fe=50.96 from the present study. This turning is due to a maximized negative interaction between Fe and Si in the liquid solution. The calculation results from the assessment of Miettinen and Vassilev-Urumov [33] agree well with the experimental data at wt.%Fe<64 but deviate largely from the higher-Fe data. This discrepancy has been successfully resolved based on the present thermodynamic optimization using the MQM, as shown in Figure 12. 

**Figure 13:**
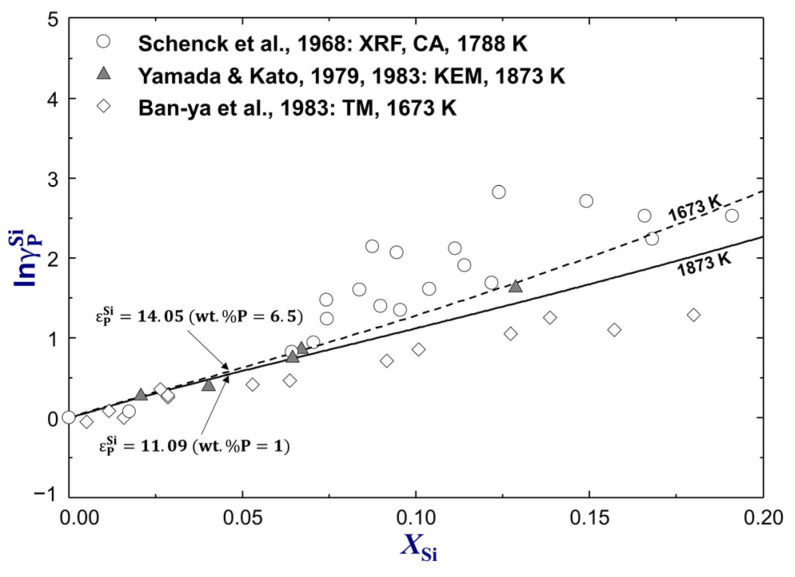
Effect of Si on the activity coefficient of P ($\gamma_{P}^{Si}$) in molten Fe–Si–P alloys from 1673 K to 1873 K, compared to experimental data [78,79,80]. For abbreviations, see Abbreviations Section.

Figure 13 shows the calculated natural logarithm activity coefficient of P affected by Si ($ln\gamma_{P}^{Si}$) in Fe-based Fe–Si–P liquid solution at 1673 K and 1873 K compared to experimental data [78,79,80]. Yamada and Kato [78] investigated the activity coefficient of P in various molten Fe–Si–P alloys at 1873 K using the Knudsen effusion method. The Si content ranged from 1wt.% to 7wt.%, but the P content was maintained at 1wt.% for all the samples. As a result, the activity coefficient interaction parameter was determined to be $\varepsilon_{P}^{Si}=11.9\pm 0.6$ at 1873 K. Ban-ya et al. [79] carried out transportation experiments to measure the vapor pressure of phosphorus above the Fe–Si–P melts (wt.%P=6.2~13.2) at 1673 K and calculated $\varepsilon_{P}^{Si}=7.68\pm 0.44$ for this temperature. The experimental results of Ban-ya et al. [79] were not favored because they assumed only P_2_(g) in the gas phase. However, the vaporization of Fe and the formation of other gas species, such as P(g) and P_4_(g), at such conditions cannot be neglected. According to the present calculations, the partial pressure of Fe(g), P_2_(g), P(g), and P_4_(g) are ${2.11\times 10}^{-6}$ atm, ${2.84\times 10}^{-6}$ atm, ${3.79\times 10}^{-8}$ atm and ${1.55\times 10}^{-12}$ atm respectively at wt.%P=9, $wt.\%Si=6(x_{Si}=0.105)$ and 1673 K. Schenck et al. [80] studied thermodynamic behavior of P in high-P Fe–Si–P alloys (wt.%P=14.6~29.8) equilibrated with the P_2_(g) gas at 1788 K using the X-ray fluorescence and chemical analysis. They proposed an average of $\varepsilon_{P}^{Si}=14.2$ for 1788 K based on the vapor pressure data, which are significantly scattered, as shown in Figure 13, and not considered in the present thermodynamic modeling. The experimental data of Yamada and Kato [78] were adopted to determine the thermodynamic properties of the Fe–Si–P liquid solution. $\varepsilon_{P}^{Si}$ was calculated to be 11.09 at 1873 K and 14.05 at 1673 K from the optimized Fe–Si–P database. 

#### 3.3.3. Predicted Phase Diagram of the Si–Fe–P System

**Figure 14:**
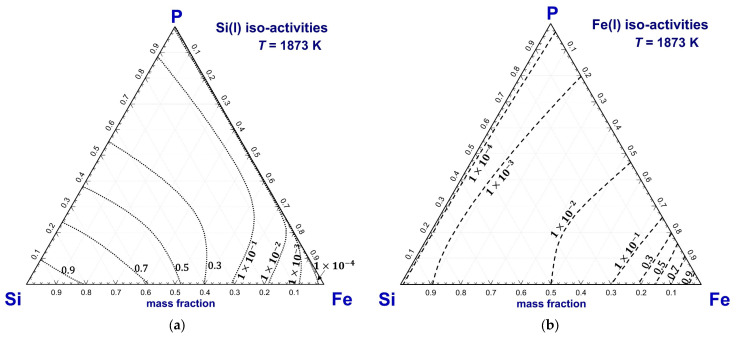

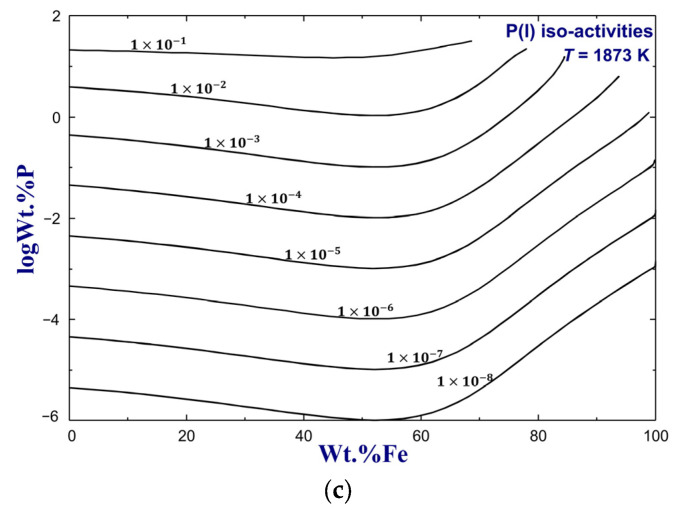
Predicted iso-activity contours of (**a**) Si(l), (**b**) Fe(l), and (**c**) P(l) of the Si–Fe–P liquid solution at 1873 K.

Based on the optimized model parameters for the Si–Fe–P system, the iso-activity contours of Si(l), Fe(l), and P(l) in pure liquid standard state at 1873 K are predicted in Figure 14. As can be seen in the figure, the iso-activities at ${1\times 10}^{-4}$, ${1\times 10}^{-3}$, ${1\times 10}^{-2}$, ${1\times 10}^{-1}$, 0.3, 0.5, 0.7, and 0.9 are plotted for Si(l) and Fe(l) and the iso-activities at ${1\times 10}^{-8}$, ${1\times 10}^{-7}$, ${1\times 10}^{-6}$, ${1\times 10}^{-5}$ ${1\times 10}^{-4}$, ${1\times 10}^{-3}$, ${1\times 10}^{-2},$ and ${1\times 10}^{-1}$ for P(l).

## 4. Summary

The Si–P and Si–Fe–P systems in the entire composition range were thermodynamically modeled based on the critical evaluation of all available experimental data. The liquid phases and solid solutions were described using the Modified Quasichemical Model (MQM) and Compound Energy Formalism (CEF), respectively. The liquid solution, solid Si, SiP, and SiP_2_ were reoptimized to resolve the discrepancies left in previous assessments of the Si–P system. Moreover, the Gibbs energies of Me_3_P, Me_2_P, and MeP solid solutions with substitution of P in Fe_3_P, Fe_2_P, and FeP with Si, respectively, and FeSi_4_P_4_ compound were well determined to reproduce the phase diagram data more accurately. According to the present optimization, a consistent and accurate thermodynamic database of the Si–Fe–P system has been constructed and used to predict unexplored thermodynamic properties and phase diagrams. The present database can be applied to process optimization of Si refining and alloy design. 

## Figures and Tables

**Table 1:** Optimized model parameters for the Si–Fe–P system. Heat capacity $C_{P}$ (J/(mol·K)), standard enthalpy of formation ${∆H}_{298.15K}^{{}^{\circ}}$ (J/mol), standard entropy $S_{298.15K}^{{}^{\circ}}$ (J/(mol·K), adjustable interaction parameter *L* (J/mol), Gibbs energy *G*, *g*, ∆g (J/mol), Curie temperature $T_{C}$ (K), magnetic moment β (μ_B_/mol).

Phase	Model Parameters
Liquid (Si, Fe, P)	$Z_{\mathrm{FeFe}}^{\mathrm{Fe}}=Z_{\mathrm{SiSi}}^{\mathrm{Si}}=Z_{\mathrm{PP}}^{P}=6$ [28,29]
	$Z_{\mathrm{PFe}}^{P}=Z_{\mathrm{PSi}}^{P}=Z_{\mathrm{FeSi}}^{\mathrm{Fe}}=Z_{\mathrm{SiFe}}^{\mathrm{Si}}=Z_{\mathrm{SiP}}^{\mathrm{Si}}=6$ [28,29,30] $Z_{\mathrm{FeP}}^{\mathrm{Fe}}=3$ [28] $\Delta g_{\mathrm{FeP}}=-56902+6.569T+(5481+3.033T)X_{\mathrm{FeFe}}-(11966-2.51T)X_{\mathrm{FeFe}}^{2}-9623X_{\mathrm{PP}}$ [28] $\Delta g_{\mathrm{SiP}}=-8812+2.092T-2343X_{\mathrm{SiSi}}+6276X_{\mathrm{PP}}$ [*] $\Delta g_{\mathrm{FeSi}}=-33710+2.26T-(12552-5.02T)X_{\mathrm{FeFe}}-(8368-4.81T)X_{\mathrm{FeFe}}^{2}-(3054-6.49T)X_{\mathrm{SiSi}}$ [29] $g_{\mathrm{FeSi}(P)}^{001}=-3849+5.86T$ [*], $g_{\mathrm{FeSi}(P)}^{011}=14770$ [*], $g_{\mathrm{FeSi}(P)}^{101}=-34095+15.06T$ [*]
	“Toop-like” interpolation with Fe as an asymmetric component [*]
FCC_A1 (Si, Fe, P)_1_(Va)_1_	$G_{\mathrm{Fe}:\mathrm{Va}}^{\mathrm{FCC}}=G_{\mathrm{Fe}(\mathrm{FCC})}^{{}^{\circ}}$, $G_{\mathrm{Si}:\mathrm{Va}}^{\mathrm{FCC}}=G_{\mathrm{Si}(\mathrm{FCC})}^{{}^{\circ}}$, $G_{P:\mathrm{Va}}^{\mathrm{FCC}}=G_{P(\mathrm{FCC})}^{{}^{\circ}}$ [*] $L_{\mathrm{Fe},P:\mathrm{Va}}^{\mathrm{FCC}}=-139787+6.49T$ [28] $L_{\mathrm{Si},P:\mathrm{Va}}^{\mathrm{FCC}}=0$ [*] $L_{\mathrm{Fe},\mathrm{Si}:\mathrm{Va}}^{\mathrm{FCC}}=-115254-2.19T-(84777-44.33T)\left(x_{\mathrm{Fe}}-x_{\mathrm{Si}}\right)+20007{\left(x_{\mathrm{Fe}}-x_{\mathrm{Si}}\right)}^{2}$ [29] $T_{C\mathrm{Fe}:\mathrm{Va}}=-201$, $\beta_{\mathrm{Fe}:\mathrm{Va}}=-2.1$ [41]
BCC_A2 (Si, Fe, P)_1_(Va)_3_	$G_{\mathrm{Fe}:\mathrm{Va}}^{\mathrm{BCC}\text{\_}A2}=G_{\mathrm{Fe}(\mathrm{BCC})}^{{}^{\circ}}$, $G_{\mathrm{Si}:\mathrm{Va}}^{{\mathrm{BCC}}_{-}A2}=G_{\mathrm{Si}(\mathrm{BCC})}^{{}^{\circ}}$, $G_{P:\mathrm{Va}}^{{\mathrm{BCC}}_{-}A2}=G_{P(\mathrm{BCC})}^{{}^{\circ}}$ [*]
	$L_{\mathrm{Fe},P:\mathrm{Va}}^{{\mathrm{BCC}}_{-}A2}=-203476+15.48T+33472\left(y_{\mathrm{Fe}}-y_{P}\right)$ [28]
	$L_{\mathrm{Si},P:\mathrm{Va}}^{{\mathrm{BCC}}_{-}A2}=-52300$ [*]
	$L_{\mathrm{Fe},\mathrm{Si}:\mathrm{Va}}^{{\mathrm{BCC}}_{-}A2}=-154014+32.29T-(63511-13.25T)(x_{\mathrm{Fe}}-x_{\mathrm{Si}})+35728{\left(x_{\mathrm{Fe}}-x_{\mathrm{Si}}\right)}^{2}$ [29]$T_{C\mathrm{Fe},P:\mathrm{Va}}=-285$ [28], $T_{C\mathrm{Fe},\mathrm{Si}:\mathrm{Va}}=504(y_{\mathrm{Fe}}-y_{\mathrm{Si}})$ [29] $T_{C\mathrm{Fe}:\mathrm{Va}}=1043$, $\beta_{\mathrm{Fe}:\mathrm{Va}}=2.22$ [41]
BCC_B2 (Si, Fe, P)_0.5_(Si, Fe, P)_0.5_(Va)_3_	$G_{\mathrm{Fe}:\mathrm{Si}:\mathrm{Va}}^{{\mathrm{BCC}}_{-}B2}=G_{\mathrm{Si}:\mathrm{Fe}:\mathrm{Va}}^{{\mathrm{BCC}}_{-}B2}=-20930$ [29] $G_{\mathrm{Fe}:\mathrm{Fe}:\mathrm{Va}}^{{\mathrm{BCC}}_{-}B2}=G_{\mathrm{Si}:\mathrm{Si}:\mathrm{Va}}^{{\mathrm{BCC}}_{-}B2}=0$ [29] $G_{P:P:\mathrm{Va}}^{{\mathrm{BCC}}_{-}B2}=G_{\mathrm{Fe}:P:\mathrm{Va}}^{{\mathrm{BCC}}_{-}B2}=G_{P:\mathrm{Fe}:\mathrm{Va}}^{{\mathrm{BCC}}_{-}B2}=G_{\mathrm{Si}:P:\mathrm{Va}}^{{\mathrm{BCC}}_{-}B2}=G_{P:\mathrm{Si}:\mathrm{Va}}^{{\mathrm{BCC}}_{-}B2}=0$ [*] $L_{\mathrm{Fe},\mathrm{Si}:\mathrm{Si}}^{{\mathrm{BCC}}_{-}B2}=L_{\mathrm{Fe},\mathrm{Si}:\mathrm{Fe}}^{{\mathrm{BCC}}_{-}B2}=L_{\mathrm{Fe}:\mathrm{Fe},\mathrm{Si}}^{{\mathrm{BCC}}_{-}B2}=L_{\mathrm{Si}:\mathrm{Fe},\mathrm{Si}}^{{\mathrm{BCC}}_{-}B2}=0$ [29]$L_{\mathrm{Fe},P:P}^{{\mathrm{BCC}}_{-}B2}=L_{\mathrm{Fe},P:\mathrm{Fe}}^{{\mathrm{BCC}}_{-}B2}=L_{\mathrm{Fe}:\mathrm{Fe},P}^{{\mathrm{BCC}}_{-}B2}=L_{P:\mathrm{Fe},P}^{{\mathrm{BCC}}_{-}B2}=0$ [28]
	$L_{\mathrm{Si},P:P}^{{\mathrm{BCC}}_{-}B2}=L_{\mathrm{Si},P:\mathrm{Fe}}^{{\mathrm{BCC}}_{-}B2}=L_{\mathrm{Si}:\mathrm{Si},P}^{{\mathrm{BCC}}_{-}B2}=L_{P:\mathrm{Si},P}^{{\mathrm{BCC}}_{-}B2}=0$ [*] $L_{\mathrm{Fe},\mathrm{Si}\text{:}P:\mathrm{Va}}^{{\mathrm{BCC}}_{-}B2}=L_{P:\mathrm{Fe},\mathrm{Si}\text{:}\mathrm{Va}}^{{\mathrm{BCC}}_{-}B2}=-3870$ [*]
Diamond_A4 Si (Si, Fe, P)_1_(Va)_1_	$G_{\mathrm{Si}:\mathrm{Va}}=G_{\mathrm{Si}(\mathrm{diamond}\text{\_}A4)}^{{}^{\circ}}$ [29], $G_{\mathrm{Fe}:\mathrm{Va}}=G_{\mathrm{Fe}(\mathrm{FCC})}^{{}^{\circ}}+1000$ [29] $G_{P:\mathrm{Va}}=G_{P(\mathrm{white})}^{{}^{\circ}}+67100-2T$ [*]$L_{\mathrm{Si},P:\mathrm{Va}}^{\mathrm{Diamond}\text{\_}A4}=-50208$ [*] $L_{\mathrm{Si},\mathrm{Fe}:\mathrm{Va}}^{\mathrm{Diamond}\text{\_}A4}=113001-0.5T$ [29]
Me_3_P (Fe)_3_(P, Si)_1_	$G_{\mathrm{Fe}:P}^{{\mathrm{Me}}_{3}P}=G_{{\mathrm{Fe}}_{3}P}^{{}^{\circ}}$ [28] $G_{\mathrm{Fe}:\mathrm{Si}}^{{\mathrm{Me}}_{3}P}=3G_{\mathrm{Fe}(\mathrm{BCC})}^{{}^{\circ}}+G_{\mathrm{Si}(\mathrm{Diamond}\text{\_}A4)}^{{}^{\circ}}-94500$ [*] $L_{\mathrm{Fe}:P,\mathrm{Si}}^{{\mathrm{Me}}_{3}P}=0$ [*]
Me_2_P (Fe)_2_(P, Si)_1_	$G_{\mathrm{Fe}:P}^{{\mathrm{Me}}_{2}P}=G_{{\mathrm{Fe}}_{2}P}^{{}^{\circ}}$ [28] $G_{\mathrm{Fe}:\mathrm{Si}}^{{\mathrm{Me}}_{2}P}=2G_{\mathrm{Fe}(\mathrm{BCC})}^{{}^{\circ}}+G_{\mathrm{Si}(\mathrm{Diamond}\_A4)}^{{}^{\circ}}-116480+24T$ [*] $L_{\mathrm{Fe}:P,\mathrm{Si}}^{{\mathrm{Me}}_{2}P}=0$ [*]
MeP (Fe)_1_(P, Si)_1_	$G_{\mathrm{Fe}:P}^{\mathrm{MeP}}=G_{\mathrm{FeP}}^{{}^{\circ}}$ [28] $G_{\mathrm{Fe}:\mathrm{Si}}^{\mathrm{MeP}}=G_{\mathrm{Fe}(\mathrm{BCC})}^{{}^{\circ}}+G_{\mathrm{Si}(\mathrm{Diamond}\text{\_}A4)}^{{}^{\circ}}-87350+17T$ [*] $L_{\mathrm{Fe}:P,\mathrm{Si}}^{\mathrm{MeP}}=14644$ [*]
FeP_2_ (Fe)_1_(P)_2_	$\Delta H_{298.15K}^{{}^{\circ}}=-191100$, $S_{298.15K}^{{}^{\circ}}=51.05$ [28] $C_{P}=77.52563+0.009348T-443846T^{-2}-1.1\times 10^{-6}T^{2}$ [28]
SiP (Si)_1_(P)_1_	$\Delta H_{298.15K}^{{}^{\circ}}=-64000$ [*], $S_{298.15K}^{{}^{\circ}}=34.78$ [*] $C_{P}=38.343+0.010878T-565000T^{-2}$ [30]
SiP_2_ (Si)_1_(P)_2_	$\Delta H_{298.15K}^{{}^{\circ}}=-79950$ [*], $S_{298.15K}^{{}^{\circ}}=64$ [*] $C_{P}=67+0.0171T$ [30]
Fe_2_Si (Fe)_2_(Si)	$\Delta H_{298.15K}^{{}^{\circ}}=-53889.7$ [29], $S_{298.15K}^{{}^{\circ}}=106.39$ [29] $C_{P}=2C_{P}({\mathrm{Fe}}_{\mathrm{BCC}})+C_{P}({\mathrm{Si}}_{\mathrm{Diamond}\_A4})$ [29]
Fe_5_Si_3_ (Fe)_5_(Si)_3_	$\Delta H_{298.15K}^{{}^{\circ}}=-234740$ [29],$S_{298.15K}^{{}^{\circ}}=209.1$ [29] $C_{P}=180.3069+0.085912T-1060722T^{-2}+2.665\times 10^{-7}T^{2}$ [29] β=2.32 [29], $T_{C}=360$ [29], P=0.28 [29]
FeSi (Fe)_1_(Si)_1_	$\Delta H_{298.15K}^{{}^{\circ}}=-76410$ [29], $S_{298.15K}^{{}^{\circ}}=46.024$ [29] $C_{P}=48.5666+0.01472T-428220T^{-2}-1.7511\times 10^{-6}T^{2}$ [29]
FeSi_2_ (Fe)_1_(Si)_2_	$\Delta H_{298.15K}^{{}^{\circ}}=-96940.44$ [29], $S_{298.15K}^{{}^{\circ}}=55.48$ [29] $C_{P}=79.02985-0.0181469T-999009T^{-2}-1.782\times 10^{-6}T^{2}$ [29]
Fe_3_Si_7_ (Fe)_3_(Si)_7_	$\Delta H_{298.15K}^{{}^{\circ}}=-247842.42$ [29], $S_{298.15K}^{{}^{\circ}}=207.3$ [29] $C_{P}=214.2176+0.10993T-2345707T^{-2}-2.3033\times 10^{-6}T^{2}$ [29]
FeSi_4_P_4_ (Fe)_1_(Si)_4_(P)_4_	$\Delta H_{298.15K}^{{}^{\circ}}=-334600$ [*], $S_{298.15K}^{{}^{\circ}}=175$ [*] $C_{P}=C_{P}({\mathrm{Fe}}_{\mathrm{BCC}})+4C_{P}({\mathrm{Si}}_{\mathrm{Diamond}\text{\_}A4})+4C_{P}(P_{\mathrm{White}})$ [*]

* optimized in the present study.

**Table 2:** Summary of the crystal structure information of all solid phases of the Si–Fe–P system.

Phase	Structure	Prototype	Space Group	Pearson Symbol
FCC_A1	Cubic	Cu	$\overset{-}{F}$ *m* $\overset{-}{3}$ *m*	*cF4*
BCC_A2	Cubic	W	*Im* $\overset{-}{3}$ *m*	*cI2*
BCC_B2	Cubic	CsCl	*Pm* $\overset{-}{3}$ *m*	*cP8*
Diamond_A4 Si	Cubic	C(dia.)	*Fd* $\overset{-}{3}$ *m*	*cF8*
Me_3_P	Tetragonal	Ni_3_P	*I* $\overset{-}{4}$	*tI32*
Me_2_P	Hexagonal	Fe_2_P	*P* $\overset{-}{6}$ *2m*	*hP9*
MeP	Orthorhombic	MnP	*Pnma*	*oP8*
FeP_2_	Orthorhombic	FeS_2_	*Pnnm*	*oP6*
SiP	Orthorhombic	SiP	*Cmc2_1_*	*oS24*
SiP_2_	Orthorhombic	GeAs_2_	*Pbam*	*oP24*
Fe_2_Si	Cubic	CsCl	*Pm* $\overset{-}{3}$ *m*	*cP2*
Fe_5_Si_3_	Hexagonal	Mn_5_Si_3_	*P6_3_/mcm*	*hP16*
FeSi	Cubic	FeSi	*P2_1_3*	*cP8*
FeSi_2_	Orthorhombic	FeSi_2_	*Cmca*	*oC48*
Fe_3_Si_7_	Tetragonal	Fe_3_Si_7_	*P4/mmm*	*tP3*
FeSi_4_P_4_	Triclinic	FeSi_4_P_4_	*P1*	---
White P	Cubic	P_4_	*I* $\overset{-}{4}$ *3m*	C*8
Red P	---	P	---	C*66

**Table 3:** Invariant reactions of the ternary Si–Fe–P system with experimental data.

Code	Invariant Reactions	Wt.%Fe	Wt.%Si	Wt.%P	*T*, K
**E1**	$Liquid=MeP+{Me}_{2}P+FeSi(s)$	71.93	9.90	18.17	1438 [*]
		71.00	14.00	15.00	1439 [71]
**E2**	$Liquid=FeSi(s)+{FeSi}_{4}P_{4}(s)+MeP$	48.58	29.38	22.04	1366 [*]
		47.10	34.00	18.90	1368 [71]
**E3**	$Liquid=FeSi(s)+{FeSi}_{4}P_{4}(s)+{Fe}_{3}{Si}_{7}$(s)	43.37	38.99	17.64	1371 [*]
		46.00	36.40	17.60	1369 [71]
**E4**	$Liquid=SiP(s)+{FeSi}_{4}P_{4}(s)+Si$	2.55	61.63	35.82	1400 [*]
		6.00	61.50	32.50	1389 [71]
**E5**	$Liquid=SiP(s)+{FeSi}_{4}P_{4}(s)+{SiP}_{2}$(s)	3.92	38.57	57.51	1428 [*]
**E6**	$Liquid={Me}_{2}P+{Me}_{3}P+BCC\_B2$	84.93	7.84	7.23	1335 [*]
**E7**	$Liquid={Fe}_{2}Si(s)+{Me}_{2}P+BCC\_B2$	81.01	14.19	4.80	1358 [*]
**E8**	$Liquid=RedP+{SiP}_{2}(s)+{FeP}_{2}(s)$	0.075	0.005	99.92	852 [*]
**U1**	$Liquid+Si={Fe}_{3}{Si}_{7}(s)+{FeSi}_{4}P_{4}$(s)	34.87	47.28	17.85	1383 [*]
**E_X_**	$Liquid=Si+{Fe}_{3}{Si}_{7}(s)+{FeSi}_{4}P_{4}(s)$	33.50	49.20	17.30	1386 [71]
**U2**	$Liquid+FeSi(s)={Me}_{2}P+{Fe}_{2}Si(s)$	80.03	15.21	4.76	1365 [*]
**U3**	$Liquid+BCC\_A2={Me}_{3}P+BCC\_B2$	86.54	5.92	7.54	1342 [*]
U4	$Liquid+MeP=FeP_{2}(s)+{FeSi}_{4}P_{4}(s)$	39.22	12.97	47.81	1410 [*]
**U5**	$Liquid+{FeSi}_{4}P_{4}(s)={SiP}_{2}(s)+{FeP}_{2}(s)$	19.77	5.32	74.91	1336 [*]

* optimized in the present study.

## Data Availability

The data presented in this work are available upon request from the authors.

## Abbreviations

AOM-Anodic Oxidation Method; BMM-Bourdon Manometer Method; CA-Chemical Analysis; DTA-Differential Thermal Analysis; ESM-Electrolytic Separation Method; HEM-Hall Measurement; ICP-MS-Inductively Coupled Plasma-Mass Spectrometry; KEM-Knudsen Effusion Method; MHM-Microhardness Measurement; MGA-Metallographic Analysis; MLE-Mass Loss Effusion; MSA-Microscopic Analysis; NAA-Neutron Activation Analysis; PN-JDM-PN-Junction Depth Measurement; RBS-Rutherford Backscattering Method; SIMS-Secondary Ion Mass Spectrometry; SMM-Static Manometer Method; SNMS-Secondary Neutral Mass Spectrometry; SRM-Sheet Resistance Measurement; TA-Thermal Analysis; TEM-Transmission Electron Microscopy; TGA-Thermogravimetric Analysis; TM-Transportation Method; TMA-Tensiometric Analysis; XRF-X-ray Fluorescence.
